# Influenza Vaccination Accelerates Recovery of Ferrets from Lymphopenia

**DOI:** 10.1371/journal.pone.0100926

**Published:** 2014-06-26

**Authors:** Nedzad Music, Adrian J. Reber, Aleksandr S. Lipatov, Ram P. Kamal, Kristy Blanchfield, Jason R. Wilson, Ruben O. Donis, Jacqueline M. Katz, Ian A. York

**Affiliations:** Influenza Division, Centers for Disease Control and Prevention, Atlanta, Georgia, United States of America; Georgia State University, United States of America

## Abstract

Ferrets are a useful animal model for human influenza virus infections, since they closely mimic the pathogenesis of influenza viruses observed in humans. However, a lack of reagents, especially for flow cytometry of immune cell subsets, has limited research in this model. Here we use a panel of primarily species cross-reactive antibodies to identify ferret T cells, cytotoxic T lymphocytes (CTL), B cells, and granulocytes in peripheral blood. Following infection with seasonal H3N2 or H1N1pdm09 influenza viruses, these cell types showed rapid and dramatic changes in frequency, even though clinically the infections were mild. The loss of B cells and CD4 and CD8 T cells, and the increase in neutrophils, were especially marked 1–2 days after infection, when about 90% of CD8+ T cells disappeared from the peripheral blood. The different virus strains led to different kinetics of leukocyte subset alterations. Vaccination with homologous vaccine reduced clinical symptoms slightly, but led to a much more rapid return to normal leukocyte parameters. Assessment of clinical symptoms may underestimate the effectiveness of influenza vaccine in restoring homeostasis.

## Introduction

Influenza viruses are common human respiratory pathogens that infect millions of people annually and cause an estimated 0.5 million deaths globally [1]–[4]. Seasonal human influenza viruses, including H3N2 and the 2009 pandemic H1N1 (H1N1pdm09) viruses, usually initiate infection in the upper respiratory tract. Clinical symptoms including fever, dry cough, sneezing, myalgia, and lethargy begin a few days after infection. In most cases, the upper respiratory tract infections are then cleared and the individual develops immunity to the specific strain of virus, although antigenic variants (“drifted” viruses) may escape this immunity to infect the same person in subsequent years. The disease caused by influenza infection is occasionally severe, especially when the virus spreads to the lower respiratory tract. As well, for reasons that are as yet unclear, influenza infection predisposes patients to secondary infection with bacteria, such as *Streptococcus pneumoniae* or *S. pyogenes* that rarely cause serious infections alone, and this superinfection is linked to increased disease severity [5]–[7].

A variety of animal models have been used to characterize the host and its immune response to infection, disease course, pathogenesis, and transmission of influenza viruses, as well as for the development of diagnostics, therapeutics, and vaccines [8], [9]. Commonly-used animal models include mice, guinea pigs, ferrets and sometimes non-human primates (NHP). Each model has advantages and disadvantages. Mice are easily housed and handled, and a large repertoire of mouse-specific reagents and transgenic and knock-out strains are available for analyzing host responses to infection or immunization. However, mice are not natural hosts for influenza virus, and human influenza viruses usually require adaptation to efficiently replicate and cause disease in mice [9]–[12], while these mouse-adapted strains may not accurately recapitulate natural infection of humans. Guinea pigs are useful models for the study of virus transmission, but show few if any clinical symptoms of infection [13]. NHP may be the most similar to humans in terms of immunological responses [14], but are expensive and difficult to handle and house.

The ferret remains the most widely accepted small animal model for influenza virus infection and vaccine protection studies [15]–[18]. Ferrets are readily infected with human and avian influenza viruses without the need for prior adaptation, and in general the course of infection in ferrets recapitulates that seen in susceptible humans. A major disadvantage to the ferret model of influenza virus infection and immunity, however, is the paucity of ferret-specific reagents available for analysis of the host response. In particular, the ability to identify leukocyte subsets is limited, making it difficult to characterize the immune response to influenza virus infection.

Several groups have begun to identify and develop antibody reagents that identify ferret leukocyte subsets [19]–[22]. We have adapted and extended previous findings in order to track ferret peripheral blood leukocyte (PBL) subsets on a daily basis following influenza virus infection. We find that, even though clinical symptoms were mild, circulating leukocyte subsets showed rapid, dynamic, and profound change in response to infection. Vaccination against influenza significantly reduced the virus-induced changes in PBL, despite only having modest effects on clinical symptoms. As well as providing a more detailed view of the inflammatory impact of influenza virus infection, these observations may help explain the protective effect of vaccination against secondary bacterial infection following influenza virus infection.

## Materials and Methods

### Ethics Statement

This study was carried out in strict accordance with Animal Welfare Act regulations by the United States Department of Agriculture (USDA) and Public Health Service Policy on Humane Care and Use of Laboratory Animals (PHS Policy) administered by the National Institutes of Health (NIH). All animal research was conducted under Centers for Disease Control and Prevention’s Institutional Animal Care and Use Committee (IACUC) approved protocol #2884YORFERC-A4: “Influenza Vaccine Development and Evaluation” in an Association for Assessment and Accreditation of Laboratory Animal Care (AAALAC) International-accredited animal facility. Animal welfare was monitored on a daily basis, and all animal handling was performed under light anesthesia (described below) and all efforts were made to minimize suffering. Humane endpoints for this study included the presentation of body weight loss exceeding 20% (relative to weight at challenge), indications of neurological symptoms, or a clinical score of 3 in any category based on the system designed by Reuman et al. [18]. However, none of the animals in this study met those criteria.

### Viruses and vaccines

Virus stocks for A/Perth/16/09 (H3N2) (Perth/16) and for A/New York/21/2009 (H1N1pdm09) (NY/21) were propagated in the allantoic cavity of 10 day-old fertile white embryonated chicken eggs (Hy-line, Mansfield, GA) at 34°C for 48 h. Virus containing allantoic fluid was harvested, aliquoted and frozen at −80°C until used in experiments. Stocks were titered in a standard plaque assay and expressed as plaque forming units (pfu) [23] using Madin-Darby Canine Kidney (MDCK) cells. The commercial 2011–2012 Fluarix trivalent inactivated influenza vaccine (TIV) (GlaxoSmithKline Biologicals, Research Triangle Park, NC) was used. This vaccine included HA and NA from A/California/07/2009 (H1N1pdm), A/Victoria/210/2009 (H3N2) and B/Brisbane/60/2008 viruses. A/Victoria/210/2009 (H3N2) virus is antigenically similar to Perth/16 virus, and A/California/07/2009 virus is antigenically similar to NY/21 virus [24]. Appropriate control antigens for serological testing were obtained from the Influenza Reagent Resource (IRR: Influenza Division, WHO Collaborating Center for Surveillance, Epidemiology and Control of Influenza, Centers for Disease Control and Prevention, Atlanta, GA).

### Ferrets, blood samples, nasal washes, and viral challenge

Male Fitch ferrets, approximately 6 months of age (Triple F Farms, Sayre, PA), serologically negative by hemagglutination inhibition (HI) assay for currently circulating human influenza H1, H3 and type B viruses, were used in this study. Prior to the initiation of the studies, all ferrets were evaluated by a licensed veterinarian and determined to be in good health and free from malformations and signs of clinical disease. During the 14 day quarantine period, animals were randomized by weight into groups. Ferrets were maintained in standard housing, provided with commercial food pellets and water, and were pair -housed during the quarantine period and after infection. Excreta pans under the cages, cage flooring, and room floors were cleaned daily.

For vaccination, ferrets were injected intramuscularly (IM) with an adult human dose (0.5 ml or 15 µg of HA) per ferret, followed by a booster vaccination 21 days later. Control ferrets were mock vaccinated with phosphate-buffered saline (PBS).

Groups of 4 ferrets (body weight: 1–1.3 kg) were infected intranasally with 1×10^6^ PFU of H1N1pdm09 (NY/21) or H3N2 (Perth/16), diluted in 0.5 ml of sterile PBS, or were mock infected with 0.5 ml of sterile egg allantoic fluid, diluted 32 µl/468 µl in PBS (to be equal to the amount of stock virus used for infection per ferret), intranasally. In the case of Perth/16, as well as naïve animals, vaccinated ferrets were infected 35 days after the initial vaccination (14 days post-boost). Baseline weights and temperatures were obtained for the three consecutive days prior to challenge and on day 0 (the day of challenge). Body temperatures were measured using an implantable subcutaneous temperature transponder (BioMedic Data Systems, Inc., Seaford, DE).

Following challenge, ferrets were monitored for changes in body weight and temperature as well as clinical signs of illness (sneezing, lethargy, nasal discharge, diarrhea and neurological dysfunction) on a daily basis for two weeks. Animals were anesthetized with a cocktail of 22 mg ketamine and 2 mg xylazine per kg of body weight, respectively, and blood samples of 200–250 µl taken from the cranial vena cava [25] on days 0, 1, 2, 3, 4, 5, 6, 7, 9, 11, and 13 relative to the day of challenge. Blood samples were collected from alternate sides each day, and care was taken to avoid hematoma formation. Nasal washes were collected with 1 ml of PBS on days 1, 2, 3, 4, 5, 6, 7, 9, and 11. Titers were determined by standard plaque assay on MDCK cells from challenge with Perth/16 or NY/21 virus.

All ferrets were euthanized on day 14 post-challenge. Animals were sedated prior to intracardiac administration of an overdose of euthanasia agent containing pentobarbital. Discomfort and distress were limited to that which was unavoidable in the conductance of the study.

### Serology

To assess antibody responses, serum was collected prior to vaccination, 21 days post-vaccination (i.e. the day of the second vaccination), on the day of viral challenge (i.e. 14 days after the second vaccine dose), and 7 and 14 days post-challenge. HI assays were performed as previously described [26]. Sera were treated with *Vibrio cholerae* neuraminidase, also known as receptor-destroying enzyme (RDE) (Denka Seiken Co. Ltd., Tokyo, Japan) and diluted to a starting dilution of 1∶10 with PBS, pH 7.2. RDE treated serum samples (25 µl) were incubated with equal volumes of inactivated viruses (4 hemagglutinating units [HAU] of A/California/07/2009 (H1N1pdm), A/Perth/16/2009, A/Victoria/210/2009 (H3N2) and B/Brisbane/60/2008 (IRR) at room temperature (RT) for 30 minutes. Then, an equal volume of 0.5% turkey red blood cells (Lampire Biological Laboratories, Pipersville, PA) was added and incubated at RT for 30 minutes. HI assays were performed in Nunc V-bottomed 96-well microtiter plates (Thermo Fisher Scientific, Rochester, NY). The HI titer was expressed as the reciprocal of the highest dilution of the samples inhibiting hemagglutination.

### Antibodies

**Figure 1:**
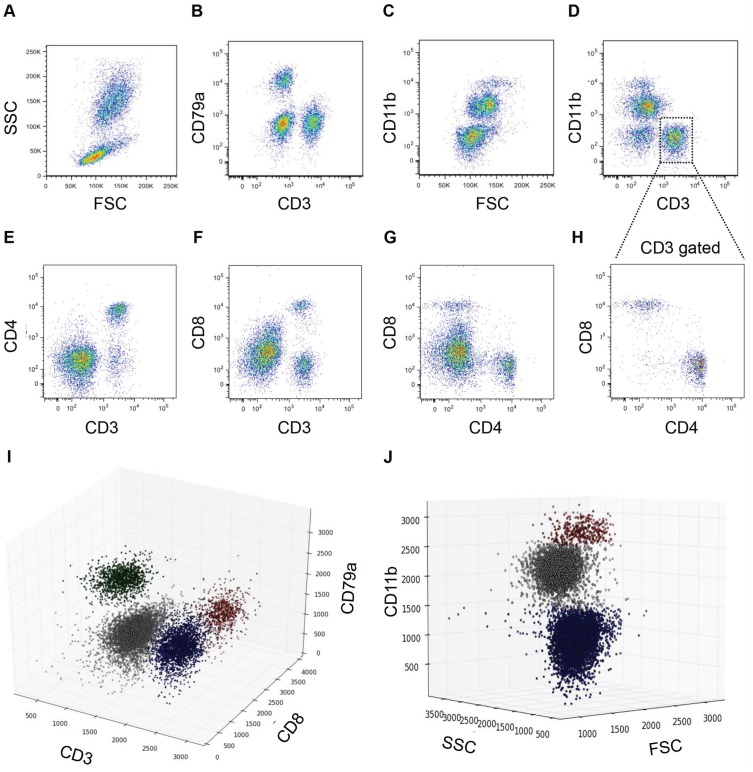
Flow cytometry of ferret peripheral blood leukocytes. Representative stains for (**A**) unstained, unpermeablized cells after gating out doublets; (**B**) CD3 vs CD79a; (**C**) CD11b; (**D**) CD3 vs CD11b; (**E**) CD3 vs CD4; (**F**) CD3 vs CD8; (**G**) CD4 vs CD8 (ungated cells); (**H**) CD4 vs CD8 (CD3 gated cells); (**I**) CD3 vs CD8 vs CD79a (B cells: Green; CD8+ve T cells: Red; CD8-ve T cells: Blue; Granulocytes/NK cells/Monocytes/DC: Gray); (**J**) FSC vs SSC vs CD11b (Monocytes/DC: Red; Lymphocytes: Blue; Granulocytes: Gray).

The antibodies used for flow cytometry, providing recognition of CD3, CD8, CD11b, and CD79a, are summarized in Table 1. While these experiments were in progress, an antibody against ferret CD4 became available and was also used in the experiments involving infection of naïve animals with Perth/16 and NY/21. Otherwise, as indicated in chart titles, CD4 cell frequency was estimated as CD3+/CD8− cells, which are almost all CD4+ (Figure 1).

**Table 1:** Antibodies used in this study.

Species/Target	Clone ID	Company	Fluor
Human CD3	PC3/188A	Santa Cruz Biotechnology, Santa Cruz, CA	FITC, AlexaFluor 647
Human CD8	OKT8	eBioscience, San Diego, CA	eFluor 450
Ferret CD4	02	Sino Biological Inc., Beijing, China	PE
Mouse CD11b	M1/70	eBioscience, San Diego, CA	FITC
Human CD79a	HM47	eBioscience, San Diego, CA	PerCP-Cy5.5
Bovine MHC class II	CAT82A	VMRD, Pullman, WA	Unconjugated

### Preparation of leukocytes

Complete blood counts were quantified from whole blood collected in EDTA Vacutainer tubes (Tyco HealthCare Group LP, Mansfield, MA) using a Hemavet HV950FS instrument per the manufacturer’s instructions (Drew Scientific, Inc., Oxford, CT). PBL were purified by hypotonic lysis of red blood cells (RBC) using erythrocyte lysing solution (0.15 M NH_4_Cl, 10 mM KHCO_3_, and 1 mM EDTA pH 7.3). One volume of fresh venous blood collected in a tube containing EDTA as anticoagulant was mixed with 69 volumes of erythrocyte lysis solution and shaken for 6 minutes at room temperature. After RBC lysis, PBL were pelleted for 10 minutes at 315×g, 4°C. The cell pellet was washed twice with flow buffer [HBSS-5+ EDTA solution (HBSS without Ca^2+^/Mg^2+^; 5% fetal bovine serum (HyClone, South Logan, UT), HEPES 10 mM, and EDTA 10 mM)], and PBL were counted, and adjusted to 2×10^6^ cells/ml in flow buffer.

Cells were labeled with surface Abs (anti-CD8, anti-CD11b and in some experiments anti-MHC class II and anti-CD4) then fixed and permeabilized using a Cytofix/Cytoperm Plus Fixation/Permeabilization Kit (BD, San Jose, CA) for intracellular staining (anti-CD3 and anti-CD79a). Isotype controls (eBioscience, San Diego, CA) were included for all antibodies. Data were acquired on a FACSCanto II flow cytometry apparatus (BD), and analyzed using FlowJo software (Tree Star, Ashland, OR).

### Statistical analysis

Statistical significance and 95% confidence intervals were calculated using a linear mixed model with repeated measures, implemented in the SAS program. Compound symmetry was used for the covariance structure for flow cytometry values; unstructured covariance was used for the HI titers. Confidence intervals were calculated using compound symmetry covariance to pool variability between groups. A p value <0.05 was used as the cutoff for statistical significance.

## Results

### Antibodies and flow cytometry

We confirmed previous results [19], [21], [22] that several antibodies produced against cell surface markers of other mammalian species cross-reacted with ferret CD3, CD8, and CD11b. In addition, we identified an antibody against human CD79a that cross-reacted with ferret CD79a (summarized in Table 1). In combination with forward and side scatter (FSC and SSC, respectively), these antibodies allowed us to identify B cells (CD79a+), T cells (CD3+), CD8 T cells (CD3+ and CD8+), granulocytes (predominately neutrophils) (CD11b moderate SSC high FSC high), and monocytes (CD11b high SSC moderate FSC high) (Figure 1). In some experiments, we also used anti-ferret CD4 (Table 1) to identify TH cells (CD3+ and CD4+) (Figure 1). In our hands, some other antibodies (such as CD25 (7D4), CD79b (CB3-1), IFN gamma (XMG1.1) and TNF alpha (MP6-XT22), previously suggested to be cross-reactive with ferret antigens, appeared to be either poorly reactive or non-specific (data not shown).

These results confirm and extend previous findings [19], [21], [22], allowing us to more comprehensively track PBL subsets in the ferret challenge model.

Normal values, based on 20–40 uninfected ferrets, are shown in Table 2 and are consistent with previous values (estimated based on cell morphology in blood smears) for neutrophils, lymphocytes, and monocytes in this species [27]. As expected for these outbred animals, there was considerable inter-animal variability in leukocyte counts (standard deviation ∼ 25–45% of average); however, for a given animal, most leukocyte counts were relatively stable over the duration of the experiments (14–49 days; standard deviation for individual animals ∼ 10–15% of average; data not shown). The exceptions were the monocyte/dendritic cell subset (SD ∼30% of average for individual animals), and CD79a+ cells (B cells) (SD ∼ 25% of average). Since we do not as yet have reagents that specifically identify ferret monocytes/dendritic cells, they were defined as CD11b-high/FSC-high, and are fairly small subpopulations that are relatively difficult to separate from granulocytes (CD11b+/FSC-moderate-high) (Figure 1C). The reason for the volatility of B cell counts is not known. In several cases, individual uninfected animals showed 2-fold or greater changes in B cell frequency over 1 or 2 days. To account for the inter-animal variability, in the experiments shown here we normalize each subset to the value observed for that individual on day 0 (i.e. immediately before infection). However, the patterns observed were very similar even without normalization (Figure S1).

**Table 2:** Normal cell values.

	CD3+ve	CD8+ve	CD4+ve	CD79a+ve	CD11b+ve	CD11b++
**Average**	29.3	5.6	17.0	13.4	37.0	3.9
**(95% CI)**	(27.1–31.5)	(4.8–6.4)	(15.2–18.8)	(11.9–14.9)	(32.8–41.1)	(3.4–4.3)
**Range**	15.3–45.2	1.9–13.7	9.5–23.4	4.6–24.5	12.9–63.3	1.7–7.9
**StDev**	7.1	2.5	4.1	4.7	13.4	1.5

Frequency of leukocyte subsets from 40 uninfected ferrets (except for CD4, for which 20 uninfected ferrets were used). Shown are the average value, the upper and lower bounds of the 95% confidence intervals, the range (lowest and highest values observed), and the standard deviation.

### Serological responses

**Figure 2:**
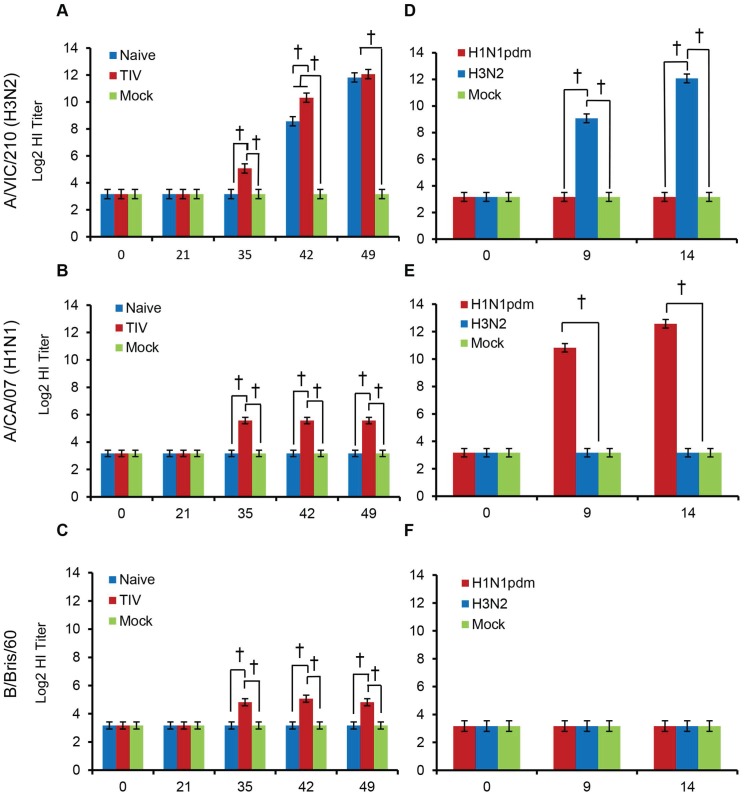
Hemagglutination inhibition (HI) titers. (**A–C**) Ferrets were immunized with commercial human TIV on days 0 and 21, and challenged with Perth/16 on day 35. Blood samples were collected on days 0, 21, 35, 42, and 49. (**D–F**) Ferrets were challenged with Perth/16 and NY/21 on day 0. Blood samples were collected on days 0, 9 and 14. HI assays were performed using (**A, D**) A/Victoria/210/2009 (H3N2), (**B, E**) A/California/07/2009 (H1N1pdm), and (**C, F**) B/Brisbane/60/2008 antigen controls. “Mock”, unvaccinated mock-infected animals; “Naïve”, unvaccinated animals challenged with Perth/16; “TIV”, vaccinated animals challenged with Perth/16; “H3N2”, unvaccinated animals challenged with Perth/16; “H1N1pdm”, unvaccinated animals challenged with NY/21. † indicates p<0.0001; * indicates 0.0001<p<0.05. Error bars represent 95% confidence intervals, calculated using compound symmetry covariance to pool variability across groups.

All ferrets were seronegative for influenza viruses before vaccination, and unvaccinated animals remained seronegative. Vaccinated animals developed modest HI titers against the three components of the vaccine (Figure 2A, B, C). Three of four vaccinated animals developed HI titers ≥40 against Perth/16 by the day of challenge (day 35 post-immunization), while the fourth animal developed a titer of 20. These titers are similar to those previously described for ferrets immunized with TIV [28]. HI titers of 40 or more are generally used as an immune correlate of protection against influenza among humans [29]–[31].

Following viral challenge, HI antibody titers to the challenge virus increased by day 7–9 post-infection and by day 14 post-challenge, GMTs had increased by greater than 150-fold over pre-challenge titers (p<0.001) (Figure 2A, D, E). This response was specific to the challenge virus, since no corresponding increase in titer was seen to the heterologous viruses (Figure 2B, C, F). Vaccinated animals showed a more rapid increase in antibody titer than naïve animals, with a significantly higher GMT at day 7 post-challenge (p = 0.024), but by 14 days post-challenge naïve and vaccinated animals had similar titers (p = 0.999) (Figure 2A). Mock-infected ferrets did not seroconvert to influenza virus.

### Clinical signs after viral challenge

**Figure 3:**
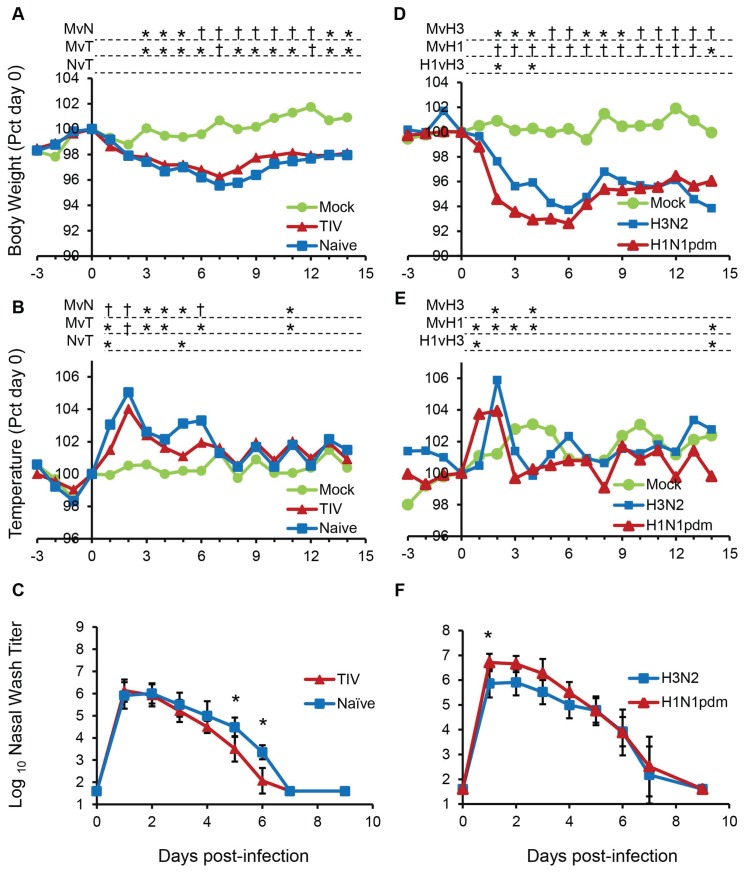
Clinical responses to infection. (**A–C**) Two weeks after booster vaccination, ferrets were challenged intranasally with 10^6^ PFU of Perth/16. (**D–F**) Unvaccinated ferrets were challenged intranasally with 1×10^6^ PFU of NY/21 or Perth/16. (**A, D**) Body weight and (**B, E**) temperature were measured daily. Data shown are normalized to the individual animals’ weight or temperature on the day of challenge (day 0). (**C, F**) Nasal washes were obtained on days 0–9 and virus titers in the nasal washes were measured. Error bars represent 95% confidence intervals. “Mock”, unvaccinated mock-infected animals; “Naïve”, unvaccinated animals challenged with Perth/16; “TIV”, vaccinated animals challenged with Perth/16. “H3N2”, unvaccinated animals challenged with Perth/16; “H1N1pdm”, unvaccinated animals challenged with NY/21. † indicates p<0.0001; * indicates 0.0001<p<0.05. “MvN”, comparing mock-infected ferrets to naïve ferrets; “MvT”, comparing mock-infected ferrets to ferrets vaccinated with TIV; “NvT”, comparing naïve ferrets to ferrets vaccinated with TIV; “MvH3”, comparing mock-infected ferrets to ferrets infected with H3N2; “MvH1”, comparing mock-infected ferrets to ferrets infected with H1N1pdm09; “H1vH3”, comparing H1N1pdm09 infected ferrets to ferrets infected with H3N2.

Ferrets were challenged intranasally with 1×10^6^ PFU of Perth/16 virus (H3N2), or with 1×10^6^ PFU of NY/21 virus (H1N1pdm09), or mock infected with the same dilution of sterile allantoic fluid in sterile PBS. Mock-infected animals did not show clinical signs, while infected ferrets all showed mild clinical signs, whether they were vaccinated or naive (Figure 3).

H3N2-infected ferrets lost between 2.2% and 8% of their body weight, with most animals beginning to regain weight after day 7 (Figure 3A, D). Vaccinated animals (range 2.2% to 5%) showed a trend towards slightly less weight loss and more rapid recovery compared to naïve ferrets (range 3.3% to 8%), but this did not reach statistical significance. Fever was observed on day 2, and on several subsequent days (Figure 3B, E). Although vaccinated and naïve animals showed similar levels of fever on most of these days, vaccinated ferrets had slightly lower body temperatures than naïve animals on days 1 and 5 (p<0.05) (Figure 3B).

Animals challenged with H1N1pdm09 showed similar, though slightly more severe, symptoms than those challenged with Perth/16 (Figure 3D–E). The mean maximum weight loss was 7.4% for animals infected with H1N1pdm09 and 6.3% for animals infected by H3N2, with H1N1pdm09-infected animals showing significantly greater weight loss than H3N2-infected ferrets on days 2 and 4. Body weights started to recover after day 7 post-challenge, but did not reach the pre-challenge (day 0) level by the end of this study (Figure 3D). H1N1pdm09-infected animals also became feverish earlier than did H3N2-infected ferrets (day 1 vs. day 2: p = 0.0072 on day 1), while on subsequent days, temperatures were similar in the groups (Figure 3E).

### Virus shedding

Virus was recovered from nasal washes after infection (Figure 3C, F). Virus shedding was observed at day 1 post-challenge for all animals and was cleared by day 7 or 9 (Figure 3C, F). Virus titers in H1N1pdm09-infected animals were higher than in H3N2-infected, although the difference was significant only on day 1 (p = 0.044) (Figure 3F). Vaccinated and naïve H3N2-infected ferrets shed similar titers of virus on the first 4 days post-infection, but vaccinated animals shed significantly lower titers by days 5 and 6 (Figure 3C). These results are similar to those previously described for ferrets infected with H1N1pdm09 and seasonal viruses [32]–[34]. Although the clinical protection conferred by TIV was modest, it is similar to that previously described for ferrets vaccinated with TIV [35].

In summary, H3N2 challenged animals exhibited only mild and temporary clinical symptoms and H1N1pdm09 challenged animals exhibited similar, but slightly more severe symptoms. Vaccination with TIV led to a moderate serological response, and a slight reduction in clinical signs and in virus shedding.

### Leukocyte subsets in vaccinated and naïve animals after H3N2 challenge

**Figure 4:**
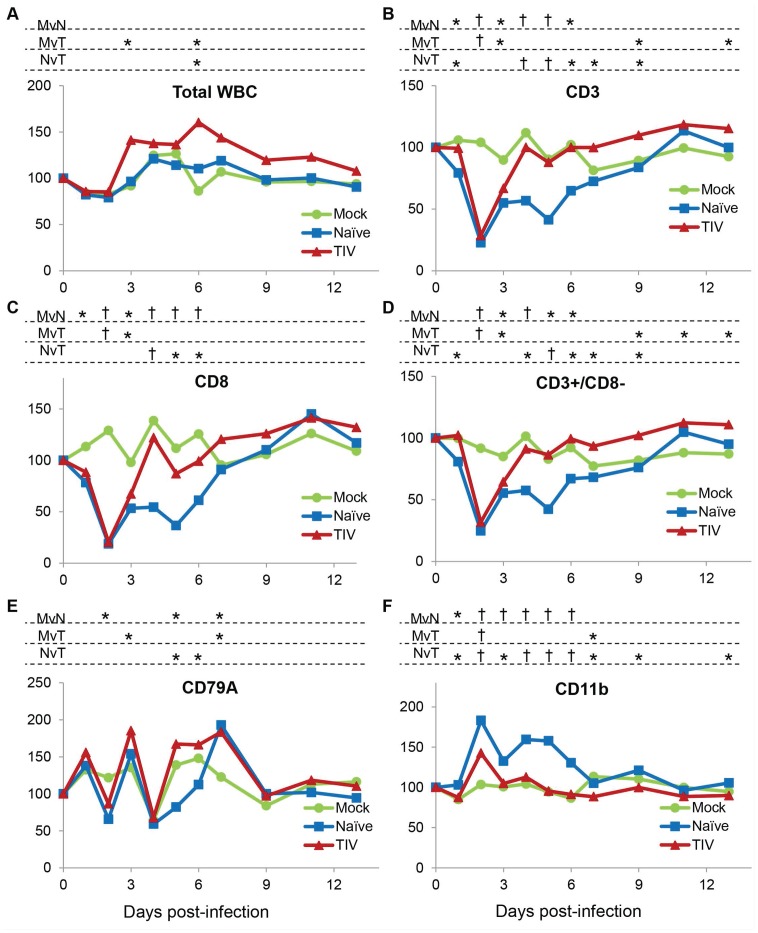
Peripheral blood leukocyte subsets following infection of vaccinated and naïve animals with Perth/16. Ferrets were bled on days 0–7, 9, 11, and 13 relative to the day of viral challenge, and cells were stained and analyzed by flow cytometry as described in the text. (**A**) Total white blood cell counts were obtained using a Hemavet apparatus. Percentages of (**B**) CD3-positive cells (T cells), (**C**) CD8-positive cells (cytotoxic T lymphocytes), (**D**) CD3-positive cells (T cells) and CD8-negative cells, (**E**) CD79a-positive cells (B cells), and (**F**) CD11b-positive cells excluding CD11b-high/FSC-high cells (granulocytes) were measured. For each animal the frequencies were normalized to the ferret’s values on day 0; the Y axis represents percent of values on day 0. “Mock”, unvaccinated mock-infected animals; “Naïve”, unvaccinated animals challenged with Perth/16; “TIV”, vaccinated animals challenged with Perth/16. † indicates p<0.0001; * indicates 0.0001<p<0.05. “MvN”, comparing mock-infected ferrets to naïve ferrets; “MvT”, comparing mock-infected ferrets to ferrets vaccinated with TIV; “NvT”, comparing naïve ferrets to ferrets vaccinated with TIV.

Total peripheral leukocyte counts remained approximately constant over the post-challenge period for all groups of ferrets, although vaccinated animals showed a small increase in total WBC counts on days 3 and 6 after infection (Figure 4A). Two days after infection, when ferrets showed peak fever, CD3+ cell counts decreased dramatically, to about 25% of pre-challenge or mock-infected levels (p<0.001) (Figure 4B). Both CD8+ (Figure 4C) and CD8- (mainly CD4+) (Figure 4D) T cells were affected, although CD8+ T cells were consistently affected more than CD8-, with some animals showing a disappearance of 90–95% of their CD8 T cells on day 2.

In both naïve and vaccinated ferrets, lymphocyte numbers rebounded significantly on the 3^rd^ day post-infection. By day 4, for the vaccinated ferrets, CD3+, CD8+, and CD3+ CD8− cells returned essentially to baseline (day 0) levels, while in naïve ferrets these cell types reached about 60% of normal levels, followed by a second drop in cell counts on days 5 and 6 (Figure 4B–D) before returning to normal levels on day 7. In vaccinated ferrets, CD3 and CD3+ CD8− counts were significantly greater than mock-infected levels (Figure 4B, D), and were significantly greater than baseline levels, from day 9 onward (Figure 4B–D). It is worth noting that this biphasic pattern of inflammation, as seen in temperature response (Figure 3B, E), has long been observed in ferrets infected with influenza [36].

B cell (CD79a+) numbers also showed a marked drop 2 days after infection, to 50–70% of mock-infected levels (Figure 4E). However, B cell numbers in mock-infected animals also dropped on day 4 and showed significant variability on several other days post-challenge.

In contrast to the lymphocyte populations, granulocyte (CD11b+; presumably predominately neutrophils) numbers increased post-infection, with a peak on day 2 post-infection in both vaccinated and naïve animals (Figure 4F). For vaccinated animals, CD11b+ counts returned essentially to normal levels after day 2, whereas for naïve animals, after a partial recovery on day 3, there was a second peak in CD11b+ cell counts on days 4 and 5, with numbers returning to normal from day 7 on. This granulocytosis was not simply a relative increase due to the loss of lymphocytes, but was an absolute increase in the numbers of cells (Figure S1F).

The monocyte/dendritic cell lineage also showed rapid and marked changes, although relatively large fluctuations were also seen in the mock-infected group of ferrets (Figure S1E). A transient early increase in the cells on days 1–2 was followed by a return to normal levels on day 3, followed by a second increase on days 5–6. However, these changes were not statistically significant when normalized to day 0 (data not shown).

The changes in lymphocytes and granulocytes during the first three days post-infection were not only rapid and marked, these changes were very consistent among animals, with each ferret showing very similar duration and extent of change in cellularity (Figure S2). Following these highly consistent changes in early infection, lymphocyte and granulocyte numbers showed more variation between individuals on subsequent days, though clear trends were readily detected (Figure S2).

In summary, although clinical signs were relatively mild and transient, changes in leukocyte subsets were rapid and dramatic. Transient lymphopenia affected both CD8+ and CD3+/CD8− T cells, with CD8+ T cells being more affected. Vaccinated animals exhibited less marked changes in most subsets, and their leukocyte subsets showed a faster return to normal levels than for unvaccinated ferrets.

### Leukocyte subsets in naïve animals after H3N2 and H1N1pdm09 challenge

**Figure 5:**
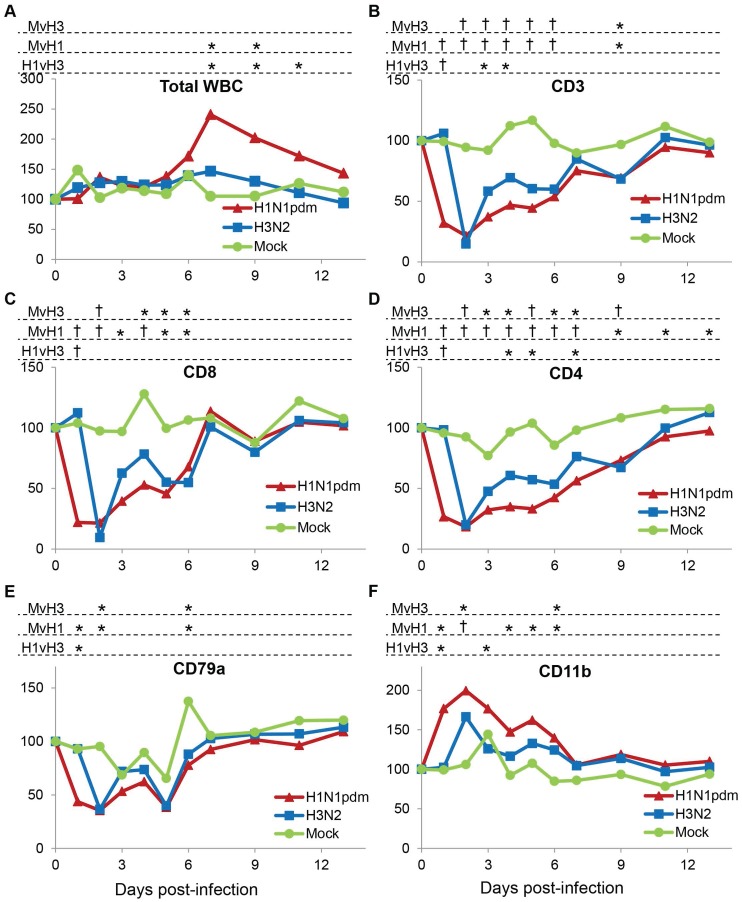
Peripheral blood leukocyte subsets following infection of naïve animals with Perth/16 and NY/21. Ferrets were bled on days 0–7, 9, 11, and 13 relative to the day of viral challenge, and cells were stained and analyzed by flow cytometry as described in the text. (**A**) Total white blood cell counts were obtained using a Hemavet apparatus. Percentages of (**B**) CD3-positive cells (T cells), (**C**) CD8-positive cells (cytotoxic T lymphocytes), (**D**) TH cells (CD3+ and CD4+), (**E**) CD79a-positive cells (B cells), and (**F**) CD11b-positive cells excluding CD11b-high/FSC-high cells (granulocytes) were measured. For each animal the frequencies were normalized to the ferret’s values on day 0; the Y axis represents percent of values on day 0. “Mock”, unvaccinated mock-infected animals; “H3N2”, unvaccinated animals challenged with Perth/16; “H1N1pdm”, unvaccinated animals challenged with NY/21. † indicates p<0.0001; * indicates 0.0001<p<0.05. “MvH3”, comparing mock-infected ferrets to ferrets infected with H3N2; “MvH1”, comparing mock-infected ferrets to ferrets infected with H1N1pdm09; “H1vH3”, comparing H1N1pdm09 infected ferrets to ferrets infected with H3N2.

We compared the effects of H1N1pdm09 infection on PBL subsets to that of H3N2. Total WBC counts remained approximately constant over the post-challenge period for all three groups of ferrets, although H1N1pdm09-infected animals showed a significant increase in total WBC counts on days 7 and 9 after infection (Figure 5A). H3N2-infected animals showed very similar responses in this experiment as in the previous experiment (Figure 4). CD3+ T cells as well as CD8+ and CD4+ cells dropped markedly on day 2 post-infection, with the reduction being most dramatic for CD8+ cells. These cell types recovered partially on days 3 and 4, followed by a second reduction on days 5 and 6, after which the cells returned to similar levels as mock-infected (Figure 5B–D). B cells showed a similar though less marked pattern, although again even mock-infected animals showed significant variability (Figure 5E). CD11b+ granulocytes increased significantly on day 2 post-infection, returned to approximately base-line levels, and were again significantly higher than mock-infected levels on day 6 (Figure 5F).

As with clinical symptoms, PBL subset changes in H1N1pdm09-infected animals were similar to those in H3N2-infected, but generally more marked. In particular, while H3N2-infected animals showed little change until day 2 post-infection, H1N1pdm09-infected animals showed significant changes in all subsets examined on the first day after challenge. Whereas H3N2-infected animals showed a very abrupt drop in T cell counts followed by rapid, if partial, recovery, the H1N1pdm09-infected ferrets had a longer trough and a less complete recovery for several days. Interestingly, CD8+ cells, as in H3N2-infected animals, showed a second drop on day 5, though to a lesser extent than in H3N2-infected ferrets. Similarly, in H1N1pdm09-infected animals, granulocyte counts peaked on day 2 post-infection, but remained elevated until day 6. Nevertheless, most PBL subsets returned to approximately normal levels by about day 7 post-infection (Figure 5).

Thus the changes within all subsets in H1N1pdm09-infected animals were similar to those in H3N2-infected, but generally more marked, and H1N1pdm09-infected animals showed significant changes in all subsets examined on the first day after challenge.

## Discussion

Ferrets are considered the most appropriate model animal for the study of human influenza disease and immunity, because they can be infected directly with human virus isolates and generally show similar clinical signs and disease severity to humans infected with the similar viruses [15]–[18]. Ferrets are large enough that serial blood samples of ∼250 µl, sufficient to perform flow cytometric analysis, can be taken daily without depleting blood volume or cellularity. Thus the ferret offers a relevant animal model where the precise infectious dose, time post-infection, and exposure history to influenza can be controlled. However, unlike humans or mice, few ferret-reactive antibodies have been available to identify specific leukocyte subsets in this species. Here, we used a panel of species cross-reactive antibodies to measure the effect of influenza virus infection on ferret leukocyte subsets. We found that infection with human influenza viruses that induce only mild and temporary clinical signs resulted in dramatic and rapid alterations in leukocyte counts and ratios. These changes are similar to, but generally more dramatic than, those described in human infections with influenza viruses. This may reflect the relatively high dose of virus used to infect ferrets, but it is also possible that the extent of these early, transient changes in leukocytes may be underestimated in human studies because of differences in timing of presentation relative to infection. The limited number of studies in mice (which are difficult because daily bleeds are not possible in this species, meaning that groups of animals must be sacrificed on each day) also indicate marked leukopenia following influenza virus infection [37].

Mock-infected ferrets were treated and handled the same as infected ferrets including a mock challenge containing the same concentration of allantoic fluid as challenged animals, yet showed little change in most cell parameters (total WBC, RBC, CD3+, CD8+, CD11b+) over the 2-week course of the experiment. This showed that the daily handling, sedation and blood samples alone did not perturb the majority of the parameters we assessed. However, even in mock-infected animals, B cells (CD79a+) did show substantial fluctuations from day to day (e.g. from day 3 to 4, Figure 4E; similar fluctuations were seen in all experiments). It is not known whether ferret B cells are intrinsically more volatile than T cells, or whether the handling and sedation involved in blood collection, etc. (although ketamine/xylazine sedation is not to our knowledge associated with changes in blood subsets), caused these changes in B cells alone. In spite of the difficulty in establishing a baseline value, ferrets did show changes in B cell counts compared to mock-infected animals on several days (Figures 4E, 5E); however, because of the variability in these cell counts we place less emphasis on these changes than on those of more consistent leukocyte subpopulations. Nevertheless, the apparent volatility of B cell populations during the course of the experiment warrants further study.

As previously reported, [32]–[34], [38], [39], infection with 1×10^6^ PFU of the seasonal viruses Perth/16 (H3N2) or NY/21 (H1N1pdm09) causes a mild and short-lived disease in ferrets. Clinical signs included a brief and moderate rise in temperature and <10% weight loss (Figure 3A, B), with H1N1pdm09 causing a slightly more severe disease than H3N2 at this challenge dose. Nevertheless, peripheral blood leukocyte subsets showed substantial and rapid changes, with marked lymphopenia and moderate granulocytosis early in the infection followed by a gradual recovery to normal leukocyte values over about 7 days.

These changes are broadly similar to those described in humans infected with seasonal or pandemic influenza viruses, for whom moderate lymphopenia is a common finding [40]–[47]. In ferrets, although the lymphopenia was moderate on most days after infection (e.g. ∼60% of pre-challenge levels on day 5), a marked lymphopenia was present 2 days post-infection, with lymphocytes as a whole dropping to ∼20% of pre-challenge levels and CD8+ T cells in some ferrets dropping to 5% of pre-challenge levels.

Neutrophilia is occasionally seen in human influenza infection [48], though it is less common in the absence of pneumonia or bacterial co-infection [47], [49]–[51]. All infected ferrets showed granulocytosis (CD11b+ cells; presumably mainly due to neutrophilia) for several days post-infection. This granulocytosis was not simply a relative increase due to the loss of lymphocytes, but was an absolute increase in the numbers of neutrophils (Figure S1). Monocytosis is also commonly seen in influenza infection in humans [43], [44]; while infected ferrets often appeared to show some degree of monocytosis, the lack of specific markers for monocytes in these experiments and the variable levels seen in mock-infected animals made this difficult to interpret.

As shown in previous studies [38], [39], H1N1pdm09 caused a more severe infection than did H3N2. Infection with H1N1pdm09 led to more rapid weight loss and earlier onset of fever than did infection with H3N2, and 3- to 7-fold higher titers of H1N1pdm09 virus were shed in nasal washes (Figure 3). PBL subsets, too, showed evidence of more severe inflammation in H1N1pdm09, with an earlier onset of changes (day 1 vs. day 2) and a more gradual recovery to baseline levels. H1N1pdm09 infections are associated with extensive cytokine production [32]–[34] as well as with greater replication in the lower respiratory tract than H3N2 infection [39], and all these factors are probably responsible for the greater impact on PBL subsets. The molecular mechanisms causing the changes in leukocyte numbers are not clear. Since we only examined peripheral blood, migration into the respiratory tract [39] or lymphoid tissue is likely to account for at least part of the loss of lymphocytes. However, the kinetics of the previously described increase in alveolar mononuclear cells in H3N2 or H1N1pdm09 infection are apparently transient [39], which does not correlate well with all of the changes in PBL observed here. While migration to the respiratory tract may be responsible for the initial loss of peripheral lymphocytes at day 2, it seems less likely that the more prolonged reduction (to day 7) is caused solely by migration. Alternatively, cytokines such as interferon may sensitize lymphocytes to apoptosis [52]–[54] and have been associated with lymphopenia and neutrophilia [55]. Studies of cytokine expression in ferret lungs following influenza virus infection showed a spike in expression of both Type I and Type II IFNs, as well as TNFα, on day 2, followed by a return to lower levels [56], [57], which correlates with the timing of lymphocyte disappearance and recovery observed in the present study. Further analysis of leukocyte subsets in tissues will be required to better understand the kinetics of PBL changes.

For the relatively mild H3N2 virus infection, there was little clinical evidence of protection by vaccination, although most ferrets achieved a serum antibody HI titer of 40, which in humans is correlated with a 50% reduction in risk of influenza infection [31], [58]. Vaccinated and non-vaccinated animals lost similar amounts of weight, but vaccinated ferrets had a slightly shorter duration of fever and lower temperatures on several days post-infection. These results are consistent with previous observations in ferrets vaccinated with TIV [28].

In contrast, vaccinated ferrets did show significant differences in several leukocyte parameters compared to naïve animals, especially after 3 days post-challenge. In particular, in vaccinated animals T cells returned to normal levels by 4 days post-infection, and by 9 days post infection were actually slightly higher than baseline values, whereas non-vaccinated animals showed reduced T cell numbers through day 6 (Figure 4B–D). Similarly, granulocytosis was relatively mild and transient (day 2 only) for vaccinated animals, while naïve animals showed a marked and persistent granulocytosis from days 2 through 6 (Figure 4F). Some of the difference might reflect lower viral titers in vaccinated vs naïve animals, but on day 4– when PBL counts in vaccinated animals had returned essentially to normal – vaccinated animals were still shedding significant levels of virus, higher than the levels in naïve animals on day 6 (Figure 3C), when the latter still showed altered PBL levels. Possibly vaccination restricts the sites of viral replication, allowing some viral shedding while reducing systemic inflammation.

One common but poorly-understood outcome of influenza virus infection is secondary infection with bacteria, such as *Streptococcus pneumoniae* or *S. pyogenes* [5]–[7], that rarely cause serious infections alone. Vaccination against influenza also confers protection against secondary bacterial infections in animal models [6], [59], [60] and humans [61]. It is interesting to speculate that the leukocyte changes observed, especially the lymphopenia, could play a direct role in susceptibility to bacterial infection. In any case, these findings suggest that influenza vaccination may confer benefits beyond the initial clinical disease, by allowing more rapid recovery of leukocyte populations and thereby providing enhanced resistance against other pathogens in the post-infection period.

## Supporting Information

Figure S1 **Absolute counts of peripheral blood leukocyte subsets following infection with Perth/16.** Ferrets were bled on days 0–7, 9, 11, and 13 relative to the day of viral challenge, and cells were stained and analyzed by flow cytometry as described in the text. Frequencies of each subset as a percentage of total WBC, obtained by flow cytometry, were applied to total WBC counts as determined using the Hemavet apparatus. **(A)** Total white blood cells, **(B)** CD3-positive cells (T cells), **(C)** CD8-positive cells (cytotoxic T lymphocytes), **(D)** CD79a-positive cells (B cells), **(E)** CD11b-high, FSC-high cells (monocytes/dendritic cells), and **(F)** CD11b-positive cells excluding CD11b-high/FSC-high cells (granulocytes) were measured. The Y axis represents thousands of cells per µl blood. “Mock”, unvaccinated mock-infected animals; “Naïve”, unvaccinated animals challenged with Perth/16; “TIV”, vaccinated animals challenged with Perth/16. † indicates p<0.0001; * indicates 0.0001<p<0.05. “MvN”, comparing mock-infected ferrets to naïve ferrets; “MvT”, comparing mock-infected ferrets to ferrets vaccinated with TIV; “NvT”, comparing naïve ferrets to ferrets vaccinated with TIV.
(TIF)

Figure S2 **Peripheral leukocyte changes in individual ferrets following infection with A/Perth/16/2009.** Changes in leucocyte subsets after Perth/16 challenge for 15 naïve ferrets (red traces) and 9 vaccinated animals (blue traces). **(A)**, CD3+ cells (T cells); **(B),** CD8+ve cells (cytotoxic T lymphocytes); **(C)**, CD11b+ve cells excluding CD11b-high/FSC-high cells (granulocytes). Data are pooled from three independent experiments. The Y axis represents the fraction of total WBC, normalized to the ferret’s value on day 0.
(TIF)
