# Supplementary figures and images for: Influenza Vaccination Accelerates Recovery of Ferrets from Lymphopenia
Source: PLoS One. 2014 Jun 26;9(6):e100926. doi: 10.1371/journal.pone.0100926 (PMC4072694; doi:10.1371/journal.pone.0100926)

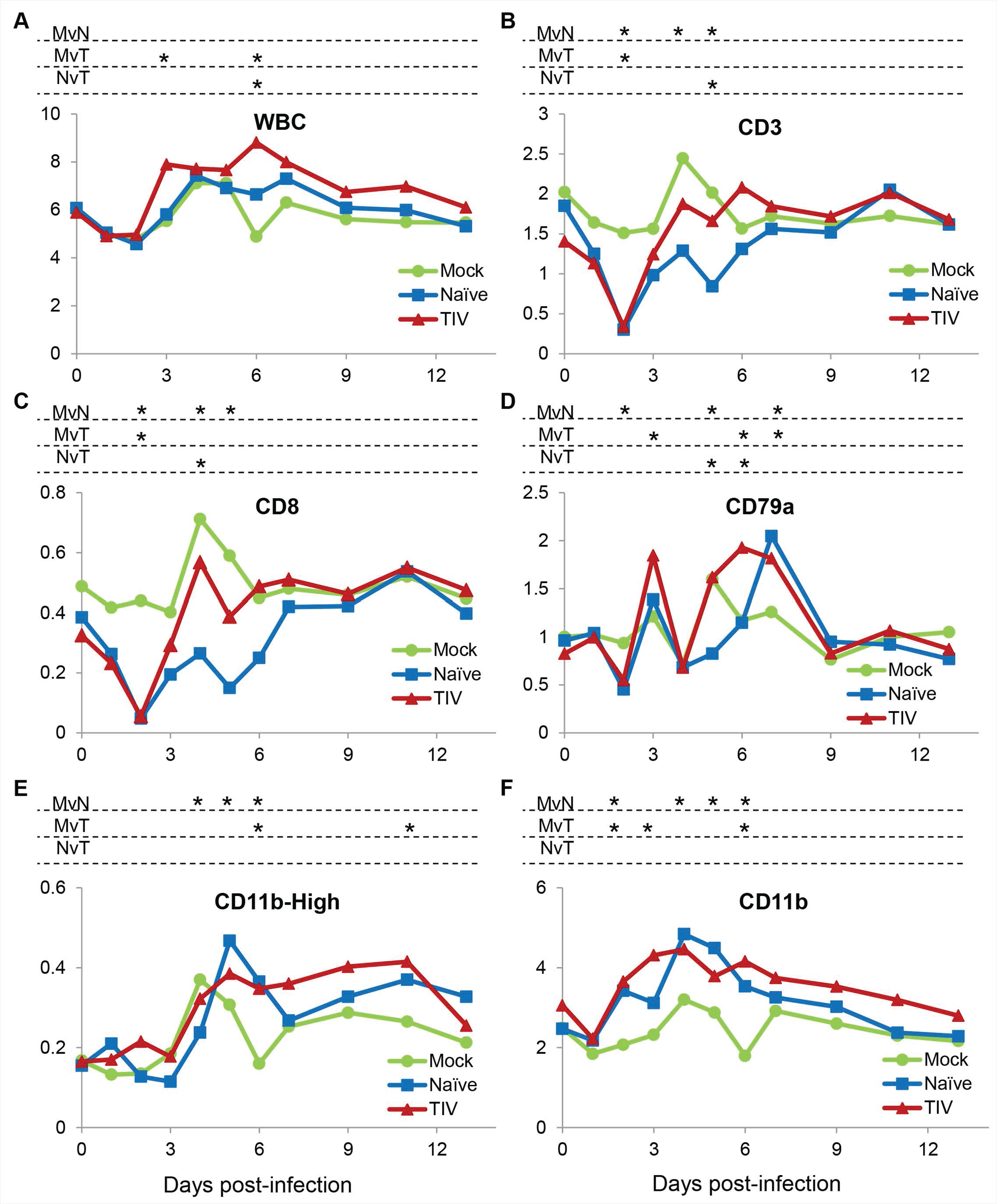

Supplement: Figure S1 — Absolute counts of peripheral blood leukocyte subsets following infection with Perth/16. Ferrets were bled on days 0–7, 9, 11, and 13 relative to the day of viral challenge, and cells were stained and analyzed by flow cytometry as described in the text. Frequencies of each subset as a percentage of total WBC, obtained by flow cytometry, were applied to total WBC counts as determined using the Hemavet apparatus. (A) Total white blood cells, (B) CD3-positive cells (T cells), (C) CD8-positive cells (cytotoxic T lymphocytes), (D) CD79a-positive cells (B cells), (E) CD11b-high, FSC-high cells (monocytes/dendritic cells), and (F) CD11b-positive cells excluding CD11b-high/FSC-high cells (granulocytes) were measured. The Y axis represents thousands of cells per µl blood. “Mock”, unvaccinated mock-infected animals; “Naïve”, unvaccinated animals challenged with Perth/16; “TIV”, vaccinated animals challenged with Perth/16. † indicates p<0.0001; * indicates 0.0001<p<0.05. “MvN”, comparing mock-infected ferrets to naïve ferrets; “MvT”, comparing mock-infected ferrets to ferrets vaccinated with TIV; “NvT”, comparing naïve ferrets to ferrets vaccinated with TIV. (TIF) [file pone.0100926.s001.tif]

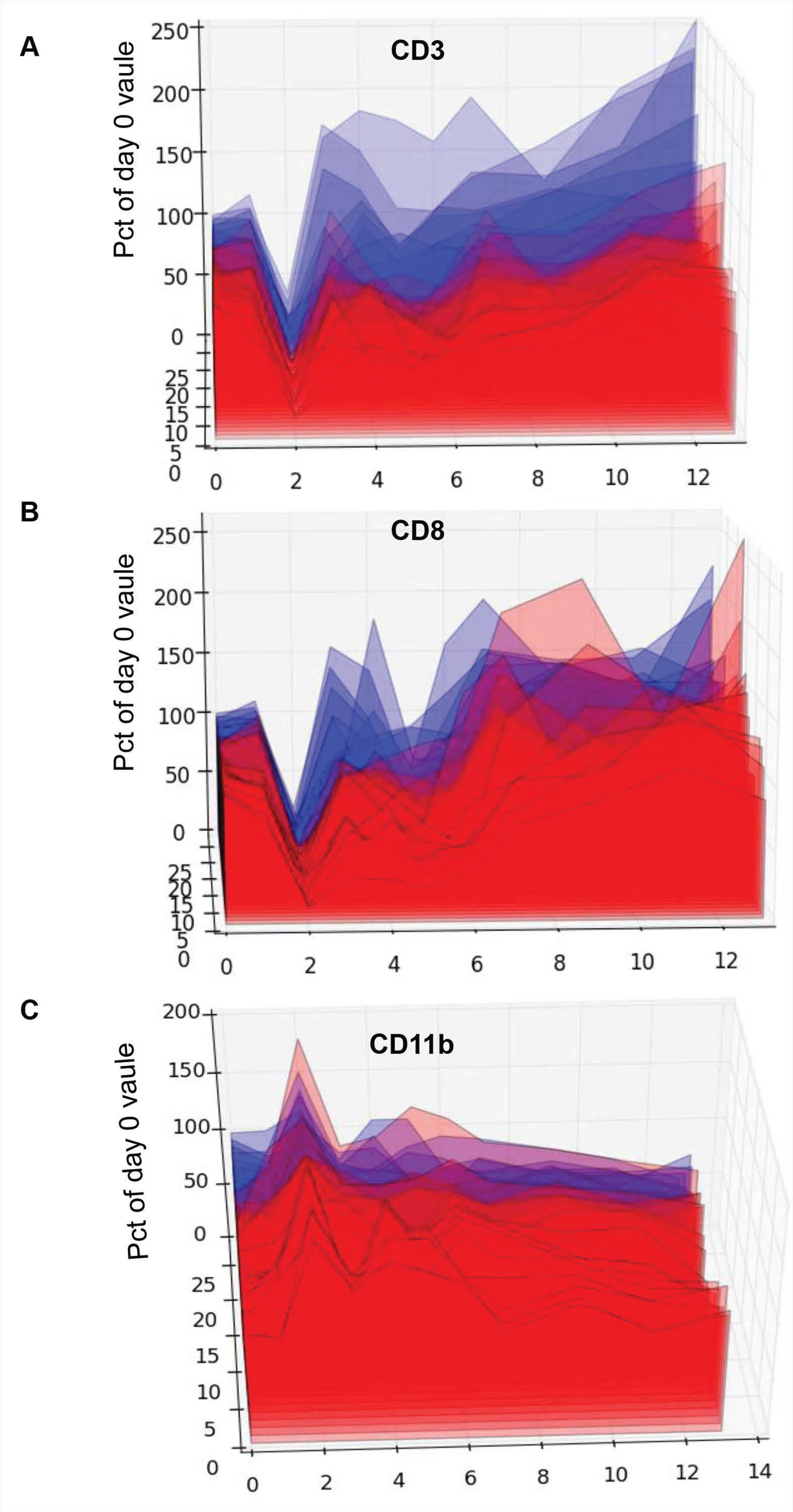

Supplement: Figure S2 — Peripheral leukocyte changes in individual ferrets following infection with A/Perth/16/2009. Changes in leucocyte subsets after Perth/16 challenge for 15 naïve ferrets (red traces) and 9 vaccinated animals (blue traces). (A), CD3+ cells (T cells); (B), CD8+ve cells (cytotoxic T lymphocytes); (C), CD11b+ve cells excluding CD11b-high/FSC-high cells (granulocytes). Data are pooled from three independent experiments. The Y axis represents the fraction of total WBC, normalized to the ferret’s value on day 0. (TIF) [file pone.0100926.s002.tif]
